# Genetic and epigenetic modifications induced by chemotherapeutic drugs: human amniotic fluid stem cells as an in-vitro model

**DOI:** 10.1186/s12920-019-0595-3

**Published:** 2019-10-28

**Authors:** Prabin Upadhyaya, Alessandra Di Serafino, Luca Sorino, Patrizia Ballerini, Marco Marchisio, Laura Pierdomenico, Liborio Stuppia, Ivana Antonucci

**Affiliations:** 1 Department of Psychological, Health and Territorial Sciences, School of Medicine and Health Sciences, “G.d’Annunzio” University, Chieti-Pescara, Via dei Vestini 31, 66013 Chieti, Italy; 2 Centre of Aging Science and Translational Medicine (Ce.S.I.-Me.T.), G. d’Annunzio University, Chieti-Pescara, Italy

**Keywords:** Human amniotic fluid stem cells, Epigenetics, Bleomycin, Etoposide, Cisplatin, BEP, microRNA, DNA methylation

## Abstract

**Background:**

Bleomycin, etoposide and cisplatin (BEP) are three chemotherapeutic agents widely used individually or in combination with each other or other chemotherapeutic agents in the treatment of various cancers. These chemotherapeutic agents are cytotoxic; hence, along with killing cancerous cells, they also damage stem cell pools in the body, which causes various negative effects on patients. The epigenetic changes due to the individual action of BEP on stem cells are largely unknown.

**Methods:**

Human amniotic fluid stem cells (hAFSCs) were treated with our in-vitro standardized dosages of BEP individually, for seven days. The cells were harvested after the treatment and extraction of DNA and RNA were performed. Real-time PCR and flow cytometry were conducted for cell markers analysis. The global DNA methylation was quantified using 5mC specific kit and promoter and CpG methylation % through bisulfite conversion and pyrosequencing. Micro- RNAs (miRNAs) were quantified with real-time qPCR.

**Results:**

The cytotoxic nature of BEP was observed even at low dosages throughout the experiment. We also investigated the change in the expression of various pluripotent and germline markers and found a significant change in the properties of the cells after the treatments. The methylation of DNA at global, promoter and individual CpG levels largely get fluctuated due to the BEP treatment. Several tested miRNAs showed differential expression. No positive correlation between mRNA and protein expression was observed for some markers.

**Conclusion:**

Cytotoxic chemotherapeutic agents such as BEP were found to alter stem cell properties of hAFSCs. Different methylation profiles change dynamically, which may explain such changes in cellular properties. Data also suggests that the fate of hAFSCs after treatment may depend upon the interplay between the miRNAs. Finally, our results demonstrate that hAFSCs might prove to be a suitable in-vitro model of stem cells to predict genetic and epigenetic modification due to the action of various drugs.

## Background

Cancer is a leading cause of death worldwide [1]. Chemotherapy constitutes the main treatment protocol for almost all tumours. Bleomycin, etoposide and cis-platinum/cisplatin (BEP) are three chemotherapeutic agents that are widely used in solo or in combination with each other (BEP regimen) or with other chemotherapeutic agents [2–4] for the treatment of various cancers [5–9], specially testicular (seminoma and non-seminoma) [10, 11] and various subtypes of ovarian cancer [12]. Each anti-cancer drug has a specific mechanism of action to arrest carcinogenesis and in particular, the exposure to these treatments causes cell death or prevent cell growth through inhibiting protein function or DNA synthesis [13–17]. There are some shreds of evidence in the literature that numerous anti-cancer drugs change the epigenetic makeup of the cells [18–21]. Studies have also examined the role of BEP on the sperm epigenome, implying the potential risk to the subsequent generations [22, 23]. The anti-proliferative effects of the different chemotherapeutic agents are key aspects contributing to tumour regression, but unfortunately, it can also affect healthy proliferative tissues, thus defining the limits of the treatment with these drugs. Tissue regeneration after cancer therapy is a crucial point for the preservation of human health, which mainly depends upon the survival of stem cells to replace the dead cells and tissues [24, 25]. The role of stem cells in the replacement of senescent or deteriorated cells of the human body is defined by their capacity of self-renewal and multilineage differentiation. Hence, stem cells can be considered a unique in vitro model to test toxicity and treatment protocols for chemotherapeutic drugs. Human amniotic fluid stem cells (hAFSCs) have been proposed so far to be an interesting model to test specific drugs and to evaluate their efficiency on cell-types of different lineages [26–30]. The hAFSCs are defined as a peculiar class of stem cells, cultivated from second-trimester amniotic fluid with the properties in between multipotent and pluripotent stem cells and they express a range of markers typical to pluripotent stem cells [31] and primordial germ cells (PGCs) [27]. Despite intensive investigations, still, there is not sound understanding on epigenetic alterations induced by chemotherapy affecting the chromatin architecture or DNA methylation. In light of these premises, the aim of the present study was to investigate epigenetic changes induced by cisplatin, bleomycin and etoposide in hAFSCs. For this purpose, we investigated global DNA methylation, gene-specific DNA methylation including the imprinted gene H19 and microRNAs to elucidate a potential epigenetic mechanism by which chemotherapeutic drugs might alter stemness (by changing the expression of pluripotency and germline markers), cell proliferation, apoptosis and chemosensitivity.

## Materials and methods

### Isolation and culture of hAFSCs

Human amniotic fluid samples (2–3 ml) were obtained from women undergoing amniocentesis (*n* = 8) for prenatal diagnosis at 16–18 weeks of pregnancy after written informed consent. All pregnant women received detailed information about the experimental protocol, which was approved by the Ethics Committee of the University of Chieti- Pescara, Italy. hAFSC were isolated from amniotic fluid and cultured until the 5th passage as described by Antonucci et al. [28]. The cells were then seeded into T25 flasks (Thermo Scientific, MA, USA), or in 10 cm tissue culture grade plates and incubated at 37 °C in a humidified atmosphere (95%) under 5% CO2.

### MTT assay

Cell viability and dose vs response were assessed using CellTiter 96® AQueous One Solution Cell Proliferation Assay (Promega Italia s.r.l., Italy), following the Manufacturer’s Protocol. Ninety-six well plates were seeded with approximately 3000 cells/well. Counting of the cells were performed using microwell cell counter. After 24 h of incubation at 37 °C in a humidified, 5% CO_2_ incubator, the hAFSCs get attached at the bottom of the plates. Then the cells were treated with concentration gradients (0.5 μM, 1 μM, 5 μM, 10 μM, 50 μM, 100 μM, 500 μM & 1000 μM) of cisplatin, bleomycin and etoposide separately and incubated at the same incubator 24, 48 and 72 h respectively, in different plates. Preparation of drugs and their concentration gradient is reported on Additional file 1: SI1. After each time interval, 20 μl of CellTiter 96® AQueous One Solution Reagent was added into each well. The plate was incubated at 37 °C in a humidified atmosphere (95%) under 5% CO2 for 2 h and absorbance was recorded at 490 nm using readwell TOUCH™- Automatic ELISA Plate Analyser (Robonik®, India). Background absorbance was first subtracted to each data point using a set of wells containing Iscove’s Modified Dulbecco’s Medium (IMDM) only, and then a dose-response curve was generated for each drug, in order to evaluate viability with respect to time concentration of those drugs. From the graph, one suitable concentration has been determined for all the future treatments.

### Treatment of hAFSCs with BEP regimen

Once the hAFSCs in the flasks or plates almost 70–80% confluency (~ 700,000 cells/plate), they were treated separately with Bleomycin (Sigma-Aldrich by Merck, Germany), Etoposide synthetic (≥98%, powder; Sigma-Aldrich by Merck, Germany) and Cisplatin (European Pharmacopoeia Reference Standard; Sigma-Aldrich by Merck, Germany) for 7 days with IC_5_ values of the respective drugs. The treatment was performed in between 4th to 6th passage. We decided to treat the cells with least (IC_5_) concentration for longer period of the time (7 days) so that the drugs do not violently kill the cells and we retain the sufficient number of cells with the drug-induced stable genetic and epigenetic changes. The preparation of the solutions for all the three drugs is described in supplementary data. The cells were incubated with the drugs for the 7 days in the incubator using the same conditions as mentioned above. The cells were observed visually with the help of an inverted binocular microscope. The old medium was removed and fresh medium in the control and medium with respective concentrations of the drugs has been added in every 2 days. Treated samples do not proliferate much and do not reach 100% confluency; however, the cells in the control (without treatment) were split once they reached 100% confluency.

### Harvesting the cells

The cells were harvested after 7 days using the standard trypsinisation protocol. 1X trypsin (Carlo Erba Reagents, Italy) and 1X Dulbecco’s phosphate-buffered saline (PBS) was used for trypsinisation and washing the cells, respectively. After harvesting, the cells with each treatment and control were divided into three equal parts. The cells in the first two parts were used for extraction of DNA and RNA, respectively whereas the whole live cells in third part were used for flow cytometry and protein analysis.

### Extraction of DNA and RNA

Total DNA was extracted with MagPurix Forensic DNA Extraction Kit (MagPurix®, Zinexts Life Science, Taiwan) and the automatic DNA extractor (MagPurix®, Zinexts Life Science, Taiwan) according to the manufacturer’s protocol. The quantification of extracted DNA and was performed using the Qubit DNA assay kit (Life Technologies, ThermoFisher Scientific, MA, USA).

Total RNA was extracted using Ribospin II (GeneAll Biotechnology Co., Seoul, Korea) by following the manufacturer’s protocol. RNA quantification was done using the Qubit RNA assay kit (Life Technologies, ThermoFisher Scientific, MA, USA). Colourimetric readings for quantification of DNA and RNA were taken at Qubit 3.0 fluorometer (ThermoFisher Scientific, MA, USA).

### Global DNA methylation

Global DNA methylation quantification was performed on the nuclear DNA (100 ng) extracted using MethylFlash Methylated DNA Quantification kit (Epigentek Group Inc., NY, USA). It is important to specify that the levels of 5-mC generally account for 0.5–2% in vertebrates as reported in the manufacturer’s protocol.

### Reverse transcription and real-time PCR

Reverse transcription was performed to prepare cDNA from the mRNA present in the total RNA using RevertAid First Strand cDNA Synthesis Kit (Thermo Scientific by ThermoFisher Scientific, MA, USA) using the manufacturer’s protocol. 100 ng of input total RNA was incubated at 65 °C for 5 min with 1 μL of 100 μM oligo (dT)18 primer and nuclease-free water with a total volume of 12 μL. Then, 1 μL of RNase inhibitor, 4 μL of reaction buffer, 1 μL of reverse transcriptase and 2 μL of dNTP mix is added to the above mix and incubated at 45 °C for 1 h and 70 °C for 5 min. Thus, 20 μL of cDNA was prepared for each sample. For realtime-qPCR, highly purified salt-free primers for all the target and reference (GAPDH) genes were ordered from Eurofins genomics, Germany (Table 1). The real-time qPCR was performed using SYBR Green chemistry. The samples were analysed in technical duplicates and the total volume for each reaction was 20 μL, which comprised of 10 μL of 2X master-mix, 0.6 μL of 100 pmol/ μL forward primer, 0.6 μL of 100 pmol/ μL reverse primer, 2 μL of cDNA (~ 10 ng/ μL) and 6.8 μL of nuclease-free PCR grade water. The 96 well 0.2 μL reactions plates containing different genes were run on real-time PCR instrument (Quant Studio 5, Applied Biosystems, ThermoFisher Scientific, MA, USA) with the standard protocol (Table 2).

**Table 1:** Primers for realtime PCR

#	Gene	Forward Primer (5′ - > 3′)		Reverse Primer (5′ - > 3′)	
		Sequence	-mer	Sequence	-mer
1	*c-Kit*	CCACACCCTGTTCACTCCTT	20	TTCTGGGAAACTCCCAT TTG	20
2	*Oct-4*	CTTGCTGCAGAAGTGGGTGGAGGAA	25	CTGCAGTGTGGGTTTCGGGCA	21
3	*Sox-2*	TTGCTGCCTCTTTAAGACTAGGA	23	CTGGGGCTCAAACTTCTCTC	20
4	*c-Myc*	TCAAGAGGCGAACACACAAC	20	GGCCTTTTCATTGTTTTCCA	20
5	*Klf-4*	AAGCCAAAGAGGGGAAGACG	20	CATGTGTAAGGCGAGGTGGT	20
6	*Vasa*	CTTAGACCCAGACGAATGTATGC	23	GTTCACTTCCACTGCCACTTC	21
7	*Boll*	GCAAGAAGAGCCTTGTTAATG	21	CCTCAGAAGGTTGCAGGTATAAG	23
8	*Stella*	GCGGAGTTCGTACGCATGA	19	CCATCCATTAGACACGCAGAAA	22
9	*Dazl*	GCTCGCCTGACGCCATCTTTG	21	GCTGATGAGGACTGGGTGCTG	21
10	*Piwil-2*	TGGTTGGAGTAGGACGCTTG	20	GGGACGGTGTGCTGAAGG	18
11	*Fragilis*	GCACCCTCTACCTGAATCTG	20	AGGATGTTGTAGCACTTGGC	20
12	*Sycp-3*	TGCAGGAGTAGTTGAAGATATG	22	CTAGCATGTCCTTAAGAAGCCTGTC	25
13	*Stra-8*	AAGGACAGCGGCGTGG AC	18	CTGGCAAGCACTGAACTGGAG	21
14	*GAPDH* (Reference gene)	ACCATCTTCCAGGAGCGAGA	20	AGTGATGGCATGGACTGTGG	20

**Table 2:** Protocol for Realtime qPCR with SYBR-green chemistry

STEP	TEMPERATURE	TIME	NO. OF CYCLES
UDG pre-treatment	50 ° C	2 min	1
Initial denaturation	95 ° C	10 min	1
Denaturation	95 °C	15 s	40 cycles
Annealing and Extension (data acquisition)	60 ° C (example)	30 s	
Denaturation for melt curve	95 °C	15 s	1
Annealing and extension	60	1 min	
(data acquisition) Dissociation for melt curve	95	15 s	

### miRNA analysis

Eight miRNAs associated with pluripotency and differentiation along with one endogenous control, all purchased as Single-tube TaqMan Advanced miRNA assays (Applied Biosystems, ThermoFisher Scientific, MA, USA) were selected for the study (Table 3). cDNA was synthesized from total RNA using TaqMan Advanced miRNA cDNA Synthesis Kit (Applied biosystem, ThermoFisher Scientific, MA, USA) by following manufacturer’s protocol (Applied Biosystems, ThermoFisher Scientific, MA, USA). The kit produces cDNAs from mature miRNAs in the total RNA samples by extending the 3′ end of the mature transcript through poly (A) addition and then lengthening the 5′ end by adaptor ligation. The modified miRNAs then undergo universal reverse transcription followed by amplification to increase uniformly the amount of cDNA from all miRNAs. The prepared cDNA was diluted 1:10 in 1X TE buffer. After the preparation of cDNA, real-time PCR reaction plates were prepared using the manufacturer’s protocol (Applied Biosystems, ThermoFisher Scientific, MA, USA). The 20 μl of each PCR reaction included 10 μL of TaqMan Fast Advanced Master Mix (2x) (Applied Biosystems, ThermoFisher Scientific, MA, USA), 1 μL of TaqMan Advanced miRNA Assay (20X) (Biosystems, ThermoFisher Scientific, MA, USA), 4 μL of RNase free water and 5 μL of diluted cDNA (2.5–5 ng). The reactions were run on quant studio 5 in a 96-well optical plate at 95 °C for 20 s, 1 cycle (enzyme activation), followed by 40 cycles of 95 °C for 1 s (denaturation) and 60 °C for 20 s (annealing/extension). The Ct data were determined using default threshold settings. The threshold cycle (Ct) is defined as the fractional cycle number at which the fluorescence passes the fixed threshold. Relative quantification was calculated in terms of delta delta Ct (∆∆Ct).

**Table 3:** Selected miRNAs used in the study

miRBase ID:	Assay ID (thermo scientific)	Stem-loop Accession #	Mature miRNA Sequence
hsa-miR-372-5p	478854_mir	MI0000780	CCUCAAAUGUGGAGCACUAUUCU
hsa-mir-34a	478047_mir	MI0000268	CAAUCAGCAAGUAUACUGCCCU
hsa-miR-17-3p	477932_mir	MI0000071	ACUGCAGUGAAGGCACUUGUAG
hsa-let-7a-5p	478575_mir	MI0000060	UGAGGUAGUAGGUUGUAUAGUU
hsa-miR-449a	478561_mir	MI0001648	UGGCAGUGUAUUGUUAGCUGGU
hsa-miR-34c-5p	478052_mir	MI0000743	AGGCAGUGUAGUUAGCUGAUUGC
hsa-miR-122-3p	477874_mir	MI0000442	AACGCCAUUAUCACACUAAAUA
hsa-miR-185-5p	477939_mir	MI0000482	UGGAGAGAAAGGCAGUUCCUGA
hsa-miR-106b-5p	478412_mir	MI0000734	UAAAGUGCUGACAGUGCAGAU
hsa-miR-145-3p	477915_mir	MI0000461	GGAUUCCUGGAAAUACUGUUCU
hsa-miR-361-5p (endogenous control)	478056_mir	MI0000760	UUAUCAGAAUCUCCAGGGGUAC

### Bisulfite conversion and pyrosequencing

PCR and sequencing primers were designed using the PyroMark® assay design software version 2.0 (QIAGEN, Germany) (Table 4). Extracted DNA from all the samples were modified using BisulFlashTM DNA Modification Kit (Epigentek Group Inc., NY, USA) which is capable of modifying 200 pg to 1 μg of DNA. The kit claims to convert 99.9% of unmethylated cytosine into uracil with less than 10% loss of DNA during conversion steps. We used 100 ng of input DNA for each sample for Bisulfite conversion. The bisulfite-treated samples were then amplified by PCR (SimpliAmp, Applied Biosystems, ThermoFisher Scientific, MA, USA), using forward and reverse primers (Table 4 for PCR primers and conditions), in which one of the primers is biotinylated. We have used KAPA HiFi HotStart Uracil+ Ready Mix PCR kit (Kapa Biosystems, Roche, Switzerland) for amplification of bisulfite-converted DNA with 1 μL of converted DNA, 0.5 μL of forward and 0.5 μL of reverse primer, 12.5 μL of Kapa Master mix, and 10.5 μL of PCR grade water for each sample. The amplification cycles include initial denaturation at 95 °C for 3 min, 30 cycles of denaturation (98 °C for 20 s), annealing (always 15 s; for annealing temperatures of different genes, Table 1) and extension (72 °C for 1 min) followed by 1 cycle of final extension (72 °C for 1 min). We did electrophoresis (Major Science, CA, USA) using 1.5 μL of the post-PCR products to visualise our band of interest and to eliminate the samples showing trailing or non-specific bands. We then used Sepharose beads (sequencing beads) to purify the final PCR product using a biotin-labelled primer. The PCR product was bound to Streptavidin Sepharose HP (Diatech pharmacogenetics, Italy), and the Sepharose beads containing the immobilized PCR products were captured using the PyroMark® Q96 vacuum preparation tool (QIAGEN, Germany), and then washed with 50 mL of 70% ethanol for 5 s, denatured with 40 mL of denaturation solution (Diatech pharmacogenetics, Italy) for 5 s, and neutralized with 50 mL of wash buffer (Diatech pharmacogenetics, Italy) for 10 s. The biotinylated single-stranded PCR products were then released into a 96-well format plate (Pyro ID plate, Diatech pharmacogenetics, Italy) containing 2 μL of 100 pmol pyrosequencing primer suspended in 38 μL of annealing buffer. Annealing of the sequencing primer to the single-stranded DNA was performed by incubating the plates on a prewarmed heat block (QBD2 Grant, Wolf Laboratories, UK) at 80 °C for 2 min followed by incubation at room temperature for 10 min. After annealing, the plate was loaded into the PyroMark® Q96 instrument (QIAGEN, Germany). Appropriate amounts of enzyme, substrate, and dNTPs (all purchased from Diatech pharmacogenetics, Italy) biotin-labelled in the appropriate wells of the cartridge (Pyro ID cartridge, Diatech Pharmacogenetics, Italy) and the cartridge is carefully inserted into the instrument prior to sequencing. The software PyroMark® CpG (QIAGEN, Germany) was prepared in the meantime. Predetermined variable positions of the CpG sites were chosen for respective markers using the assay software. The software automatically generates a dispensation order of dNTPs and control dispensations based on the sequence to analyze. Control dispensations (not part of the sequence to analyze) were included in the dispensation order to check the performance of the reactions. Usually, there was one control injection at the beginning of the sequence and then approximately one for every CpG site. There was no chemiluminescence expected at these control injections and they were used to monitor the reagents and the sequence quality. Then the PyroMark® Q96 instrument was run and the analysis was performed. Following the sequencing reaction, the data collected were analyzed using the PyroMark® Q96 software for CpG methylation quantitation and the corresponding percent methylation values for each site and the data were displayed as a pyrogram. The percentage of methylation was expressed for each DNA locus as %5-mC divided by the sum of methylated and unmethylated cytosines. We tested each marker in technical duplicates and used their average in the statistical analyses.

**Table 4:** Primers and their annealing temperatures used in Pyrosequencing

Gene Promoter	Forward Primer (5′ → 3′) [Bio] = Biotinylated at 5′ end	Reverse Primer (5′ → 3′) [Bio] = Biotinylated at 5′ end	Sequence primer (5′ → 3′)	No. of CpGs assayed	No. of CpGs Analysed	Amplicon size	Annealing temperature (°C)
*C-Kit*	GGAGGGGGGAAAAAGTGTATGAAAATTTG	[Bio]TTCTACTCAATTTCTCCACCTACTT	AAATTTGGGTTTTTAGAGTAA	8	8	179	63
*Sox-2*	AGTAAGGAAGGTTTTGAGGATAGA	[Bio]ATATCATTATTCTCCCCCTCATCCACAA	AGGTTTGGGTTTTTTAAT	6	4	187	64
*Oct4*	[Bio]ATGGGGGAATTTTTTATATTTTAGAGTT	CACCACCATTAAACAAACATCC	AAAAAATTAAATAATCCCTT	10	9	373	59
*NANOG*	[Bio]TGTATTTTTAGTAGAGAGGGGGTTT	ACCCAACAACAAATACTTCTAAATTCACC	ATTCACCACCTTTCCAACTT	6	4	237	65
*H19*	GGTTTTGGAGGTTAGTGTTTT	[Bio]CTCAACCCCTAAAACTAACTTAACA	TTGTATTATTTTTTTTTTT GAGAGT	5	5	187	64

### Immunophenotyping with flow cytometry

#### Antibodies used

Primary unconjugated anti-human monoclonal antibodies (IgG) against OCT4, SOX2 and NANOG proteins, and appropriate secondary fluorophore-conjugated antibodies which bind with those IgG were used for flow cytometry. The primary monoclonal antibodies used were mouse anti-human SOX2 antibody (ThermoFisher Scientific, MA, USA), mouse anti-human NANOG antibody (ThermoFisher Scientific, MA, USA) and rabbit anti-human OCT4 antibody (ThermoFisher Scientific, MA, USA). The secondary antibodies used were Alexa Fluor® 488 conjugated goat anti-mouse IgG antibody (ThermoFisher Scientific, MA, USA) and Alexa Fluor® 532 conjugated goat anti-Rabbit IgG (H + L) cross-adsorbed antibody (ThermoFisher Scientific, MA, USA).

#### Flow cytometry analysis

hAFSCs were stained with anti-human primary antibodies, in order to analyse the expression of protein markers, with some modifications in the procedure described before [32, 33]. Briefly, 5 × 105 cells were incubated with 100 μl 20 mM ethylenediaminetetraacetic acid (EDTA) at 37 °C for 10 min and then washed. Washing buffer (phosphate-buffered saline [PBS], 0.1% sodium azide, 0.5% bovine serum albumin) was used for all of the washing steps (3 ml washing buffer, with centrifugation at 400×g for 8 min at 4 °C). To increase the permeability of the membranes for antibodies in case of intracellular staining, 1 ml Perm 2 (Becton, Dickinson and Company, Franklin Lakes, New Jersey, USA) was added to each tube and the cells were incubated for 10 min at room temperature in the dark. The samples were then washed and resuspended in 100 μl washing buffer containing the appropriate amount of primary antibody as per manufacturer’s instructions and incubated for 30 min at 4 °C in the dark. At the end of this incubation, the cells were washed thrice. If secondary antibody staining is required, the samples were re-suspended in 3% BSA/PBS containing fluorophore-labelled secondary antibody at the optimal dilution as per manufacturer’s instructions. The cells were then incubated for 30 min at 4 °C in the dark, washed thrice and fixed in 1 mL of 0.5% paraformaldehyde with 5 min incubation at room temperature. Then the cells were washed by centrifugation and resuspended in washing buffer and kept at 4 °C in the dark until analysed using a FACSCanto flow cytometer (BD Biosciences by Becton, Dickinson and Company, NJ, USA) and the FACDiva v6.1.3 software (BD Biosciences by Becton, Dickinson and Company, NJ, USA). Quality control was performed using a regular check with Rainbow Calibration Particles (BD Biosciences by Becton, Dickinson and Company, NJ, USA). Debris was excluded from the analysis by gating on the morphological parameters, and 20,000 non-debris events in the morphological gate were recorded for each sample. To assess the non-specific fluorescence, we used isotype controls. All of the antibodies were titrated under assay conditions and optimal photomultiplier voltages were established for each channel. The data were analysed using the FlowJoTM software (Tree Star Inc., OR, USA). The mean fluorescence intensity (MFI) ratio was calculated by dividing the MFI of positive events by the MFI of negative events.

### Statistical analysis

For individual sample, technical duplicates or triplicates were used depending upon the type of the experiment and their averages were taken for data interpretation. Such averages from 5 to 8 different hAFSC lines were obtained from each experiment and processed statistically, using Microsoft Excel 2010 (Washington, USA) and Graph Pad Prism V6 (California, USA). Dose-response time curves and respective IC values were determined using nonlinear regression. Statistical significance was determined using analysis of variance (ANOVA) and Student’s t-test, depending upon the data type. ANOVA was performed when multiple concentrations of the same drug are compared (as in dose vs viability for different concentrations). Student’s t-test was used when two data sets were compared (as in control vs bleomycin/cisplatin/etoposide) and adjusted using the Holm–Sidak correction. Data are presented as mean ± SD. *P* values were expressed as **** when *p* <  0.0001, *** when *p* <  0.001, ** when *p* <  0.01 and * when *p* <  0.05.

## Results

### Cytotoxic effects of BEP on hAFSCs

**Fig. 1:**
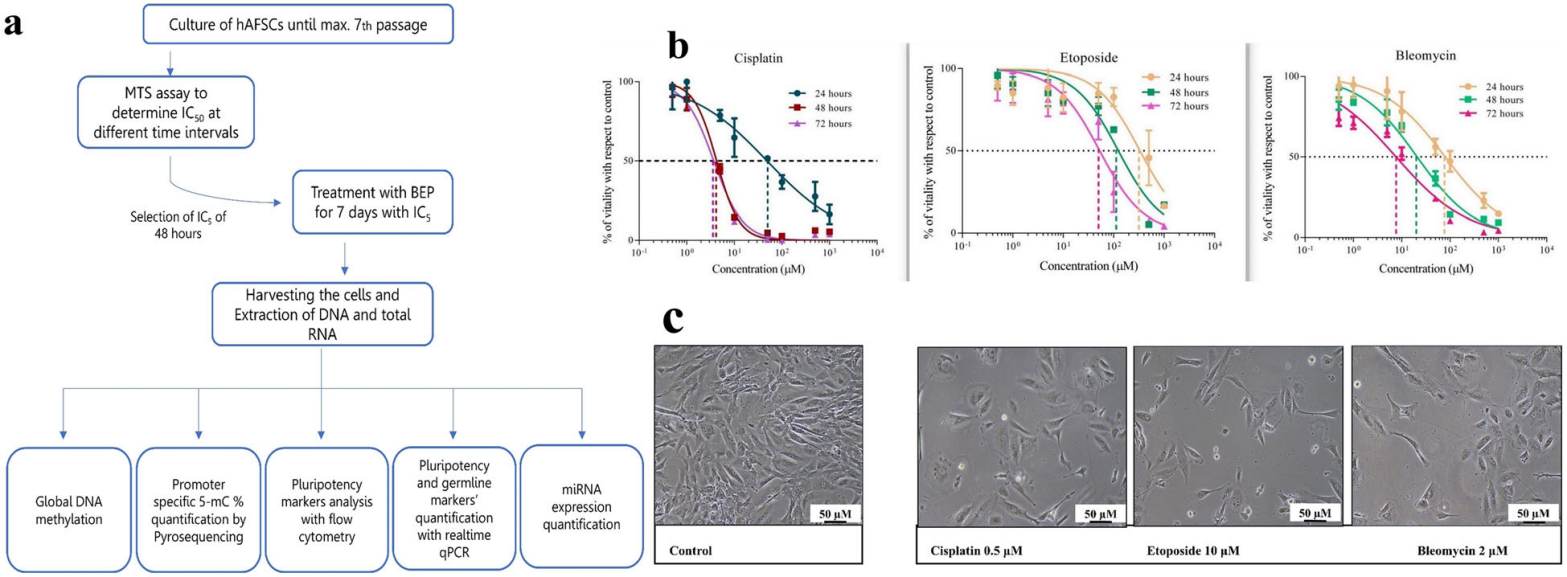
**a** Experimental design. **b** Dose-response curve determining IC50 value after BEP treatment at 24, 48 and 72 h **c** Microscopic photographs of the hAFSCs after 7 days of BEP treatment. Photos were taken under 10X magnification. Decrease in cell density in treatment with respect to control due to cytotoxic action of the drugs can be clearly seen

Treatment with concentration gradient (0.1 μM to 1000 μM) followed by 3-(4,5-dimethylthiazol-2-yl)-2,5-diphenyl tetrazolium bromide (MTT) assay suggested that hAFSCs display a significant decrease in viability in a dose and time-dependent manner when treated with cisplatin, bleomycin and etoposide (Fig. 1 b). From the dose-response curve, it was found that IC_50_ values get decreased with longer duration of the treatments (Table 5). However, the curve becomes properly sigmoidal at 48 h, suggesting it as a suitable time for short duration cytotoxicity experiments (Fig. 1 b). To get drug-induced stable genetic and epigenetic changes, we decided to treat the cells for a longer period of time (7 days). Since the cytotoxicity of the drugs increases with time, we treated the cells with a concentration 10 times more dilute (IC_5_) than IC_50_ of 48 h so that the drugs do not violently kill the cells and we retain a sufficient number of cells. The hAFSCs were therefore treated for seven days with calculated IC_5_ concentrations, i.e. 0.5 μM for Cisplatin, 2 μM for bleomycin and 10 μM for etoposide, respectively (Fig. 1 a and Table 5). Under these experimental conditions, the hAFSCs showed less viability with a toxic effect on cell density and cell growth, as documented by microscopic images (Fig. 1 c).

**Table 5:** IC_50_ and corresponding IC_5_ values for BEP at 24,48 and 72 h

	IC_50_ [μM]			*P* value^a^ (for IC50) corresponding to 24, 48 and 72 h	IC5 [μM] b		
	24 h	48 h	72 h		24 h	48 h^c^	72 h
Cisplatin	50	5	3	< 0.001	5	0.5	0.3
Etoposide	300	100	40	< 0.001	30	10	4
Bleomycin	80	20	8	< 0.001	8	2	0.8

^a^Significance was determinated by ANOVA, individually for 24, 48 and 72 h

^b^IC_5_ used were the concentration 10X dilute than IC_50_

^c^For uniformity, we have used IC_5_ of 48 h for all the drugs throughout the study

### Effect of BEP regimen on the expression of pluripotency and germ cell markers

**Fig. 2:**
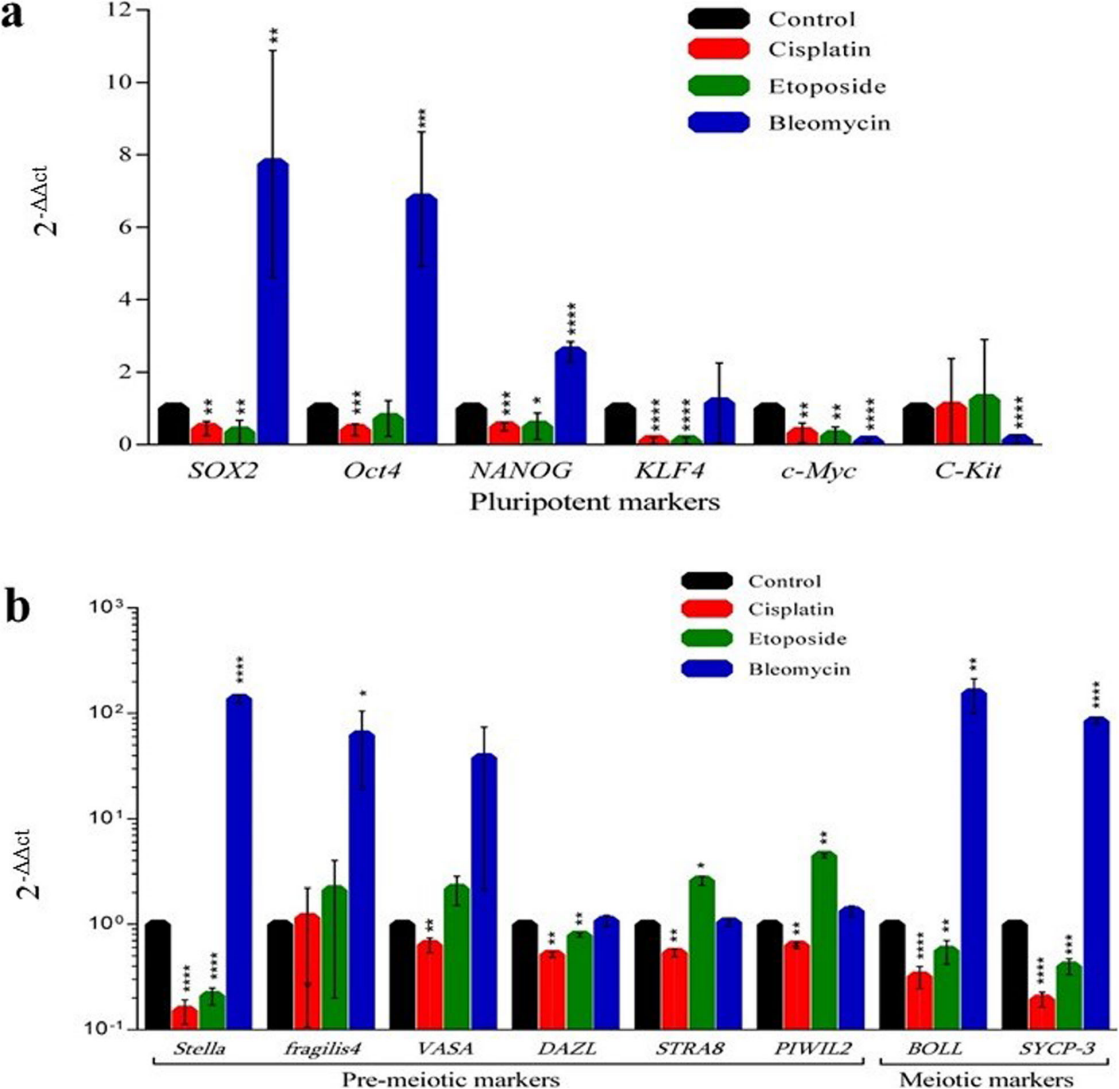
Expression of **a** Pluripotency markers *SOX2, Oct4, NANOG, KLF4, c-Myc and C-Kit* and **b** Germline markers (*Stella, fragilis, VASA, DAZL, STRA8, PIWIL2, BOLL and SYCP3*) in treated cells with respect to control. The expressions were compared with control, normalizing control expression as 1. *p < 0.05, **p < 0.01, *** *p* < 0.001 and ****p < 0.0001

Real-time PCR was performed to study the effect of anticancer drugs on a subset of pluripotency markers, as well as germ cell-specific genes, at 7 days post-treatment. hAFSCs exposed to cisplatin and etoposide presented a downregulation of pluripotency markers (*Oct4, SOX2, KLF4, c-Myc* and *NANOG*) (*P* <  0.05) while expression of the *Oct4, NANOG* and *SOX2* were upregulated in cell culture treated with bleomycin (P <  0.05), as shown in Fig. 2 a. Subsequently, markers of premeiotic (*Stella, Fragilis, Vasa, STRA8, PIWIL2, DAZL*) and meiotic (*BOLL, SCYP-3*) stages of germline cells were studied. Transcriptionally, these three drugs appear to act completely different. Notably, more or less downregulation of all germline markers was detected in cells treated with cisplatin while etoposide had induced a slight reduction in the expression of meiotic stage markers except for *STRA8* and *PIWIL2*. In contrast, bleomycin has induced the upregulation of the expression of most of the premeiotic and all meiotic genes (Fig. 2 b).

### Evaluation of pluripotency markers with flow cytometry on hAFSCs after treatment with BEP

**Fig. 3:**
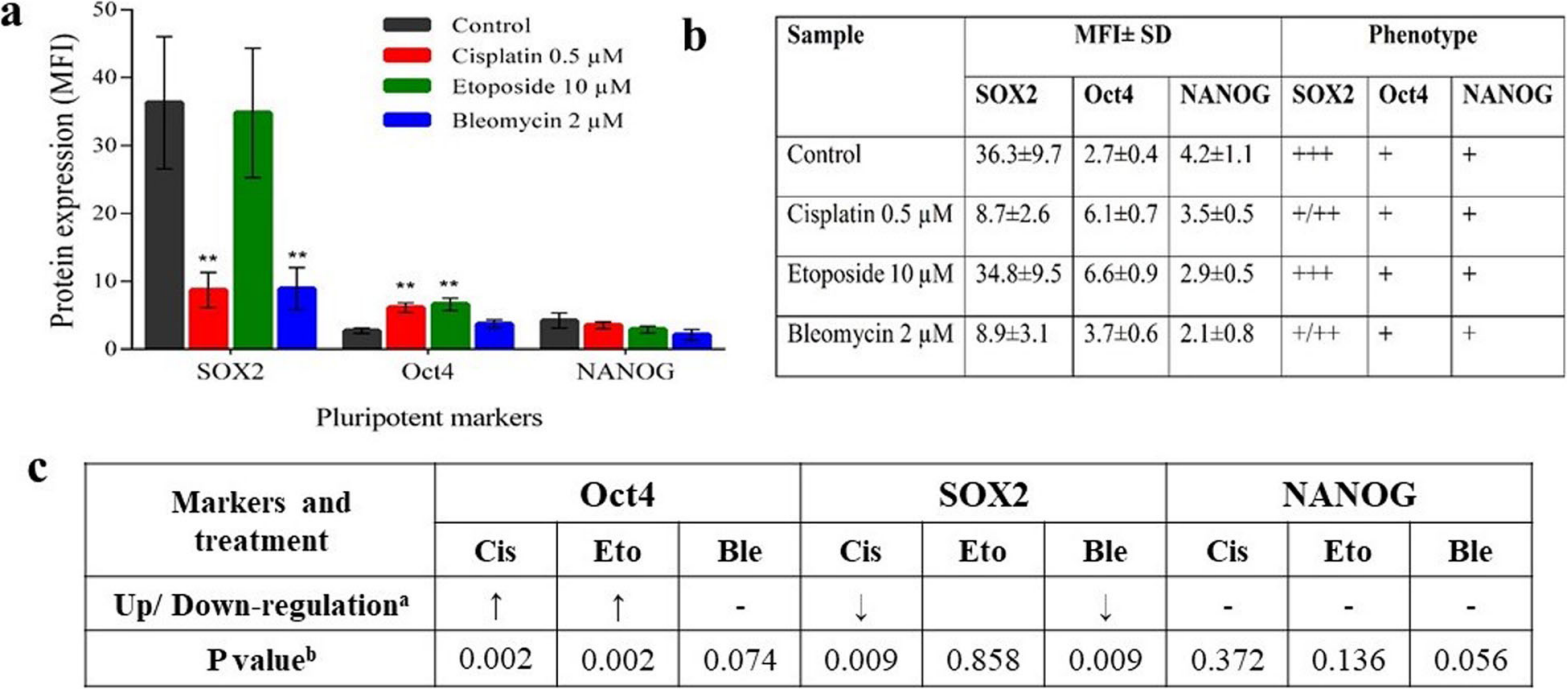
Expression of functional pluripotency markers in Flow cytometry: **a** Bar diagram and **b** Tabular representation of expression of SOX2, Oct4 and NANOG proteins in control and BEP treated samples. Expressions are shown in terms of MFI ± SD. Phenotypic classification was done based on the expression of the markers, considering the amount of mean fluorescence intensity (MFI). **c** Up or downregulation of functional pluripotency markers in BEP treatment with respect to control. Cis = Cisplatin 0.5 μM, Eto = Etoposide 10 μM, Ble = Bleomycin 2 μM. ^**a**^ Upregulation = ↑; Downregulation = ↓; ^**b**^ *P* values < 0.05 are considered significant

We used flow cytometry to assess the expression profile of the principal pluripotency markers in treated hAFSCs with BEP regimen. In Fig. 3, we have reported the mean fluorescence intensity (MFI) ratio ± SD for each gene and their respective phenotypes. Contrary to realtime qPCR data, the expression of the transcription factor Oct4 was found to be increased after the exposure of the cells to cisplatin and etoposide (Fig. 3). Conversely, the expression of SOX2, which is another transcription factor essential for the maintenance of pluripotency in stem cells, was found to be decreased during treatment with cisplatin and bleomycin, whereas it was not significantly decreased during etoposide treatment. Additionally, the expression of NANOG, an antigen associated with the maintenance of pluripotency and self-renewal was not significantly changed during any treatment (Fig. 3).

### Global and gene-specific DNA methylation profiles of hAFSCs after treatment

**Fig. 4:**
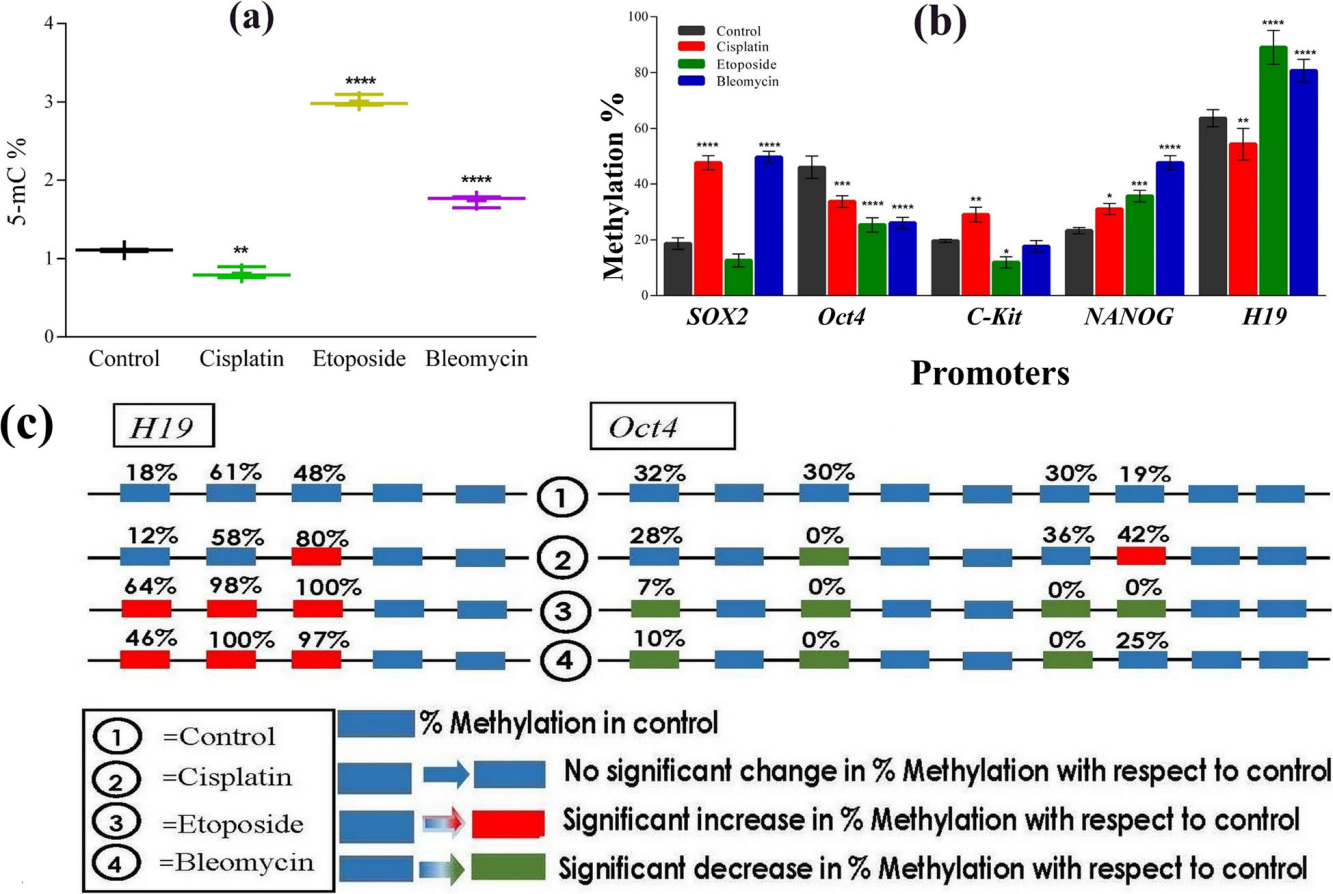
Dynamic changes in the methylation of the DNA during the treatments: Amount of methylated DNA (5-mC %) **a** in the total DNA **b** in the gene specific CpG island regions within the total DNA and **c** in the individual CpG sites in H19 and Oct4, within the CpG islands of hAFSCs. *p < 0.05, **p < 0.01, *** p < 0.001 and ****p < 0.0001

The analysis of global 5′-methylcytosine (5-mC) in the genomic DNA of treated cells showed statistically significant changes. The measured 5-mC levels ranged from 0.8 to 3% (Fig. 4 a). The averages of five biological replicates have been used as controls and for each treatment (Additional file 1: SI2). As compared to control cells, a decrease in global methylation status was observed in hAFSCs treated with cisplatin (0.8% vs 1.1%; *P* < 0.01). On the contrary, a higher percentage of methylation was found in cultures exposed to bleomycin and etoposide, 1.7 and 3.0% respectively (*P* < 0.0001) (Fig. 4 a). Concerning the methylation of specific-genes, the modifications of promoter CpG islands tend to be highly dynamic and significantly variable for each specific treatment. The methylation profiles of the candidate promoter regions were determined by pyrosequencing analysis and revealed that *SOX2, C-Kit* and *NANOG* were hypermethylated in hAFSCs treated with cisplatin (47.7, 29, 31%, respectively) compared to control (18.6, 19.6, 23.3%, respectively), with the sole exception of decreased methylation of *Oct4* after treatment (33.7% vs 46%) (Fig. 4 b). In addition, the methylation status of cells treated with etoposide exhibited a hypomethylation of *SOX2, Oct4* and *cKit* (12.7, 25.3 and 12%, respectively) except for hypermethylated *NANOG* (35.6%). Under bleomycin treatment, the promoter region of *Oct4* and *C-Kit* genes were significantly hypomethylated (26 and 17.6% respectively) while high levels of methylation were present in *SOX2* and *NANOG* (49.6 and 47.6%) (Fig. 4 b). The majority of the CpG sites were hypomethylated in all analyzed treatments. On the other hand, the methylation status of CpG islands in H19 was investigated and dynamic changes of this paternally imprinted gene were observed in all treatments., H19 hypomethylation was found in cells cultured with cisplatin with respect to control (54.3% vs 63.6%, *P* < 0.01) while hypermethylation was exhibited by hAFSCs treated with etoposide and bleomycin (89 and 80.6% respectively, *P* < 0.0001) as reported in Fig. 4 b and 4 c. It is important to emphasize that the data presented revealed that DNA methylation and gene expression are not always positively correlated (Fig. 4 b and Fig. 2). Interestingly, it has been observed that analysed CpG site 3 of *Oct4* was fully demethylated (30 to 0%) in all pharmacological exposures (Fig. 4 c). In addition to this, CpG-6 and 7 were also fully demethylated (0%) in etoposide treatment and CpG-6 in bleomycin treatment. Surprisingly, on the contrary to *Oct4*, some of the CpG sites of *H19* promoters were heavily methylated with a maximum of 100% methylation. Explicitly, analysed CpG-2 and CpG-3 were heavily hypermethylated (~ 100%) during bleomycin and etoposide treatment (Fig. 4 c).

### Dynamic changes in miRNA expression during BEP treatment

**Fig. 5:**
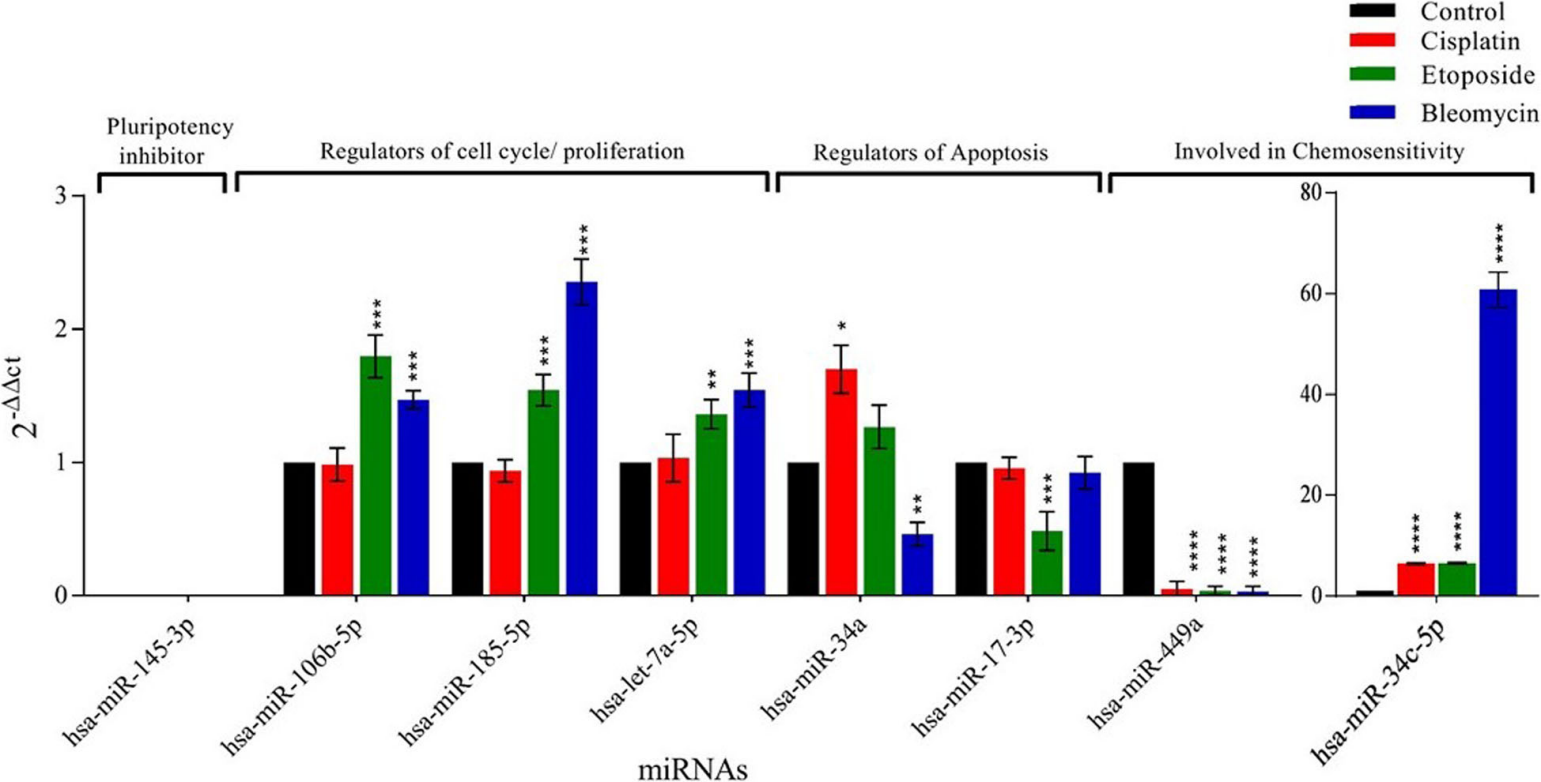
Differential expression of miRNAs in control and treated samples. miRNAs are divided based on their functions. *p < 0.05, **p < 0.01, *** *p* < 0.001 and ****p < 0.0001

In order to identify miRNA changes associated with anticancer therapy, a panel of 8 small non- coding RNAs was analyzed with the use of realtime-qPCR comparing treated cells with control. The miRNAs with a significant difference in expression (*P* < 0.5) are reported in Fig. 5 and Table 6. These miRNAs were specifically chosen as a result of detailed literature search as they are involved in a variety of biological processes (Table 6) such as: pluripotency (hsa-miR-145-3p) [34], cell cycle and cell proliferation (hsa-miR-106b-5p, hsa-miR-185-5p, let-7a-5p) [35–38], Apoptosis (hsa- miR-34a, hsa- miR-17-3p) [39, 40] and chemosensitivity (hsa-miR-34c-5p, hsa-miR-449a) [41–44] (Fig. 5). The amount of each miRNA expression and their roles are reported in the Table 6. The null expression of hsa-miR-145-3p in all treated samples and controls confirms the previous finding that hsa-miR-145 represses core pluripotency factors *Oct4, SOX2* and *KLF4* [34]. The expression of pluripotency markers in hAFSCs and absence of hsa-miR-145 suggest that the same miRNA regulates the pluripotency in hAFSCs. Also, though all the three drugs are cytotoxic, their treatment may induce opposite and dynamic expression levels for some miRNAs, like in the case of hsa- mir-34a. Moreover, Bleomycin and etoposide induce the change in expression of most of the tested miRNAs, cisplatin being the least (Fig. 5). hsa-miR-34c-5p and hsa-miR-449a were found to be up and down-regulated (*P* < 0.05), respectively in BEP treatment with respect to control samples. Notably, bleomycin and etoposide treated cells showed high expression of hsa-miR-106b-5p, hsa-miR-185- 5p and hsa-let-7a-5p, implicated in cell cycle and proliferation (P < 0.05, fold-change increase range: 1.36–2.35). The miRNAs involved in the regulation of apoptosis (hsa-miR-17-3p and miR-34a) showed variable expression in all the three treatments. In fact, miR-34a was upregulated in cisplatin and downregulated in bleomycin while hsa-miR-17-3p was downregulated only in etoposide treatment.

**Table 6:** Fold change in expression of miRNAs after treatments with cisplatin, etoposide and bleomycin with respect to control. In column 3, ‘↑’ represents significant (P < 0.05) increase; ‘↓’ represents significant (*P* < 0.05) decrease; and ‘-’ represents non-significant (P>0.05) change

1. BiologicalFunction	2. miRNA	3. Significant Fold change (p < 0.5)			4. Reports from previous studies	5. Ref.
		Cisplatin	Etoposide	Bleomycin		
Pluripotency	miR-145-3p	No expression	No expression	No expression	Represses OCT4, SOX2, and KLF4 and thus pluripotency in human embryonic stem cells	[34]
	miR-106b-5p	–	1.8 ↑	1.5 ↑	Promotes Proliferation by targeting B3G, promotes stem-cell-like phenotype	[35, 36]
	miR-185-5p	–	1.5 ↑	2.4 ↑	Inhibits cell proliferation and induces cell apoptosis by targeting VEGFA	[37]
Cell cycle and proliferation	let-7a-5p	–	1.4 ↑	1.5 ↑	High expression inhibit proliferation and induce apoptosis	[38]
	miR-34a	1.7 ↑	–	2.1 ↓	Ectopic miR-34a induced apoptosis and a cell cycle arrest in the G1-phase, by targeting p53	[39, 40]
Apoptosis	miR-17-3p	–	2.1 ↓	–	miR-17-3p is downregulated when p53 is active, thus inducing apotosis and vice versa	[40]
	miR-34c-5p	6.5 ↑	6.5↑	60.8 ↑	miR-34c-5p was downregulated in paclitaxel-resistant gastric cancer samples, MiR-34c enhances chemosensitivity of Ishikawa cell to cisplatin	[41, 42]
Chemosensitivity	miR-449a	19.7 ↓	27.3 ↓	31.6 ↓	Ectopic expression of miR-449a increased the apoptosis induced by cisplatin, miR-449a is proapoptotic and targets BCL2 expression	[43, 44]

The numbers in the column 3 represents ‘times the fold change’ after each treatment with respect to control. miRNAs are divided based on their functions. Column 4 describes their function

## Discussion

Through our experiments, we have evaluated the cytotoxicity of bleomycin, etoposide and cisplatin in hAFSCs, which showed time and dose-dependent cytotoxic effect. Epigenetically, differences in the global DNA methylation, hyper as well as hypomethylation, are observed in treated cells. We revealed a significant decrease in the 5-mC percentage in total DNA after cisplatin exposure, while a significant increase was observed after bleomycin and etoposide exposure. This result suggests that the methylation status of the genome of hAFSCs is in continuous flux during treatment. In addition, taking into consideration of our data, the chemotherapy activity could alter the pluripotency-associated genes of stem cell pool present in the adult body. These observations suggest that anti-cancer drugs can influence self-renewal and differentiation properties of stem cells. Transcriptionally, cisplatin and etoposide mediated an inhibitory effect on the expression of core pluripotency genes, concomitant with the downregulation of germline markers, particularly meiotic stage markers. On the contrary, bleomycin-induced the activation of *Oct4, NANOG* and *SOX2* and the most of premeiotic and all meiotic markers at the transcriptional level. However, interesting enough, the three tested markers such as *Oct4, NANOG* and *SOX2* at the protein level were not upregulated, suggesting towards the previously studied phenomena that although changes in mRNA and proteins are concordant for most genes, genes that are rapidly repressed upon a stimulus, can have uncoupled mRNA and protein levels [45]. It has been suggested that such differences in mRNA and protein expression occurs due to post-transcriptional, translational and protein degradation regulation [46]. We suspect epigenetically it may be regulated by some miRNAs by binding to mRNAs, which is the future scope of this study. Along with global DNA methylation, we have also explored the methylation percentage of the promoter regions of the pluripotency-related genes as potential biomarkers for chemosensitivity. The most important findings were identifying methylation patterns in *SOX2, C-Kit, Oct4* and *NANOG* genes such as the hypermethylation of DNA in the promoter of *SOX2, C-Kit* and NANOG in cisplatin treated hAFSCs, hypomethylation of CpG islands in *SOX2, Oct4* and C-Kit markers in etoposide treatment, and highly dynamic modifications of promoter CpG islands in bleomycin treated hAFSCs. Intriguingly, the study showed that the complete demethylation of CpGs 3 and 7 in the promoter region of Oct4. Recently, several studies have reported that not every CpG is able to influence gene expression with its methylation status; some CpGs are regulatory and others are not directly responsible for gene silencing [47, 48]. However further research should be conducted to understand the functional role of each single CpG. In addition, we observed the evident no correlation between DNA methylation and gene expression in some treatments for some genes (e.g. SOX2 in bleomycin treatment). This may occur when the gene expression controlling region does not fall under our tested CpG region of the promoter, but located elsewhere in the promoter, or in some other areas of the gene, or at a distal regions of the genome that influence genome activity- for example, as promoters of non-coding RNAs [49, 50]. Another epigenetic aspect of crucial importance is represented by aberrant DNA methylation of imprinted gene H19 during chemotherapy. Our data show that imprinting alterations can occur in hAFSCs during BEP treatment. The methylation status of the paternally imprinted gene fluctuated from 54.3 to 89% during treatments with the three drugs. H19 hypomethylation was found in cells cultured with cisplatin while hypermethylation was exhibited by hAFSCs treated with etoposide and bleomycin. Considering the miRNA, we found significant differential expression of the majority of miRNAs with and without treatment. An interesting finding was the absence of expression of hsa- miR-145-3p in all controls and treated hAFSCs. The null expression of hsa-miR-145-3p in all treated samples and controls confirms the previous finding that has-miR-145 represses core pluripotency factors *Oct4, SOX2* and *KLF4* [34]. Moreover, based on our analysis, we postulated that two different mechanisms are responsible for the differential expression of miRNAs:

1. *Due to the direct actions of chemotherapeutic agents on the cells*
2. *To maintain the homeostasis: (A) as defence mechanisms of the cells against the drugs and (B) to revert the changes taken at first place as direct actions of the drug(s).*

We suggested that the differential change in the expression of miR-185-5p, let-7a-5p, miR-17-3p and miR-34c-5p are due to the direct action of the drugs, and their differential expression in some or all treatment are responsible for hampered proliferation [37, 38], higher chemosensitivity [41, 42] and/or apoptosis [40] of the hAFSCs. On the other hand, hsa-miR-106b-5p and miR-449a are responsible for the cellular defence against the drugs by promoting cellular proliferation [35, 36] and chemoresistance [43, 44], respectively. The dynamic expression of hsa- mir-34a suggest both the mechanisms. Upregulation of this miRNA in cisplatin treatment suggests hsa-miR-34a induced apoptosis and a cell cycle arrest in the G1-phase, by targeting p53, whereas downregulation in bleomycin treatment suggests the opposite phenomenon [39, 40]. It is tempting to assume that the higher expression of pluripotency and PGC markers as we found in real-time qPCR analysis is probably due to the action of has-miR-34a, but it is yet inconclusive and needs further confirmations. Therefore, the success of any chemotherapeutic treatment may depend upon the proper balance between the two above mentioned phenomenon, and understanding both of these mechanisms are crucial in designing drugs with high efficacy and minimum negative effects.

## Conclusion

To our knowledge, this is the first study on genetic and epigenetic alterations carried out individually by bleomycin, cisplatin and etoposide on hAFSCs. Our study adds new findings to the present literature about the modes of action of these three chemotherapeutic agents. It is well known that these three drugs primarily target and damage cellular DNA, thus inducing apoptosis [13–17]. Taken together, it was observed that each drug could kill stem cells, significantly alter their stemness and other cell properties and bring various epigenetic changes, and this might cause the various negative effects after chemotherapy. Regarding the negative or side effects of the drugs, the decrease in stem cell pool and these epigenetic changes combined, due to chemotherapy can be the contributing factors for alopecia [51], infertility [52] and neurological impairments [53].

Though our in-vitro study is performed on a stem cell lineage, it is not erroneous to assume that chemotherapy may induce epigenetic changes to all the cell of the body to some extent. Literature suggest that the epigenetic makeup of non-stem cells gets affected as well due to the actions of cisplatin [54], etoposide [55, 56] and bleomycin [57]. Moreover, introducing other drugs and other types of body cells in such studies might help us to get more insights into epigenetic changes due to chemotherapy. This could provide us with a better understanding of cancer, its suitable treatment and management of the side effects. In addition, further investigations are still needed to clarify the epigenetic effect of the BEP regimen in different stem and non stem cell types. In summary, the results considered more important for the toxicity of the drugs are related with alterations of epigenetic machinery, and each anticancer agents showed a different effect in terms of stemness, cell growth and proliferation. Conclusively, this study evokes us to consider that other widely used chemotherapeutic drugs may have the potential to change epigenetic makeup throughout the stem cell pools of the body. However, it is important to improve our understanding of the molecular modifications underlying complex cellular mechanisms and try to consider each drug target in its full epigenetic context [58, 59].

## Supplementary information

**Additional file 1: SI1.** Drugs preparation. **SI2.** The 5-mC% values of controls and for each treatment.

## Data Availability

The sequence information about the miRNAs used in the study can be found by entering the stem loop accession number (provided in the Table 3) at miRBase (Release 22.1) repository, [http://www.mirbase.org/]. The procedure of drugs preparation can be found on supplementary information SI1. The global DNA methylation percentages across different lines of hAFSCs in control and treatments and are made available as supplementary information SI2.

## Abbreviations

∆∆Ct: delta delta Ct
5-mC: 5′-methylcytosine
ANOVA: Analysis of Variance
BEP: Bleomycin, Etoposide and Cisplatin
Ct: Threshold cycle
hAFSCs: Human Amniotic Fluid Stem Cells
IMDM: Iscove’s Modified Dulbecco’s Medium
MFI: Mean Fluorescence Intensity
miRNA: micro-RNA
MTT: 3-(4,5-dimethylthiazol-2-yl)-2,5-diphenyl tetrazolium bromide
PBS: Phosphate Buffered Saline
