# Supplementary material for: Genetic and epigenetic modifications induced by chemotherapeutic drugs: human amniotic fluid stem cells as an in-vitro model
Source: BMC Med Genomics. 2019 Oct 28;12:146. doi: 10.1186/s12920-019-0595-3 (PMC6816179; doi:10.1186/s12920-019-0595-3)
Supplement: Supplementary file 1 — Additional file 1: SI1. Drugs preparation. SI2. The 5-mC% values of controls and for each treatment. [file 12920_2019_595_MOESM1_ESM.docx]

**Additional file**

**SI1: Drugs preparation**

The drugs were freshly prepared for each treatment. For every single drug, a different stock solution was prepared and then, diluted in order to get the desired concentration of working solution. Bleomycin and Cisplatin were prepared in cell culture medium (IMDM), while Etoposide solution was prepared with 0.01% of DMSO in IMDM (*v/v).* hAFSCs were not stimulated with control solvent as DMSO at very low concentrations is not toxic for the cells [1].

[ 1. Adler S, Pellizzer C, Paparella M, Hartung T, Bremer S. The effects of solvents on embryonic stem cell differentiation. *Toxicol Vitr* 2006; **20**: 265–271.]

**SI2: The 5-mC% values of controls and for each treatment.**

|  | **Line 1**  **(5P)** | **Line 2**  **(3P)** | **Line 3**  **(3P)** | **Line 4**  **(4P)** | **Line 5**  **(4P)** | **Average (Mean±SD)** |
| --- | --- | --- | --- | --- | --- | --- |
| **Control** | 1.1 | 1.13 | 1.08 | 1.11 | 1.11 | **1.106± 0.018** |
| **Cisplatin 0.5 µM** | 0.79 | 0.9 | 0.76 | 0.81 | 0.75 | **0.802± 0.05** |
| **Bleomycin 2 µM** | 1.65 | 1.77 | 1.79 | 1.66 | 1.75 | **1.724± 0.06** |
| **Etoposide 10 µM** | 2.96 | 3.1 | 2.98 | 3.06 | 2.95 | **3.01± 0.06** |
